# NGS Panel Testing of Triple-Negative Breast Cancer Patients in Cyprus: A Study of *BRCA*-Negative Cases

**DOI:** 10.3390/cancers12113140

**Published:** 2020-10-27

**Authors:** Maria Zanti, Maria A. Loizidou, Kyriaki Michailidou, Panagiota Pirpa, Christina Machattou, Yiola Marcou, Flora Kyriakou, Eleni Kakouri, George A. Tanteles, Elena Spanou, George M. Spyrou, Kyriacos Kyriacou, Andreas Hadjisavvas

**Affiliations:** 1 Department of Electron Microscopy/Molecular Pathology, The Cyprus Institute of Neurology and Genetics, 6 Iroon Avenue, 2371 Agios Dhometios, Nicosia, Cyprus; mariaz@cing.ac.cy (M.Z.); loizidou@cing.ac.cy (M.A.L.); panagiotap@cing.ac.cy (P.P.); christinam@cing.ac.cy (C.M.); kyriacos@cing.ac.cy (K.K.); 2 Cyprus School of Molecular Medicine, The Cyprus Institute of Neurology and Genetics, 6 Iroon Avenue, 2371 Agios Dhometios, Nicosia, Cyprus; kyriakimi@cing.ac.cy (K.M.); gtanteles@cing.ac.cy (G.A.T.); georges@cing.ac.cy (G.M.S.); 3 Bioinformatics Department, The Cyprus Institute of Neurology and Genetics, 6 Iroon Avenue, 2371 Agios Dhometios, Nicosia, Cyprus; 4 Biostatistics Unit, The Cyprus Institute of Neurology and Genetics, 6 Iroon Avenue, 2371 Agios Dhometios, Nicosia, Cyprus; 5 Department of Medical Oncology, Bank of Cyprus Oncology Center, 32 Acropoleos Avenue, 2012 Strovolos, Nicosia, Cyprus; yiola.marcou@bococ.org.cy (Y.M.); florakyriakou@gmail.com (F.K.); eleni.kakouri@bococ.org.cy (E.K.); 6 Clinical Genetics Department, The Cyprus Institute of Neurology and Genetics, 6 Iroon Avenue, 2371 Agios Dhometios, Nicosia, Cyprus; elenaa@cing.ac.cy

**Keywords:** triple-negative breast cancer, germline genetic testing, NGS of gene panels, genetic predisposition to breast cancer

## Abstract

**Simple Summary:**

In Cyprus, approximately 9% of triple-negative (negative in common breast cancer receptors—estrogen, progesterone, and human epidermal growth factor receptor 2 (HER2) receptors) breast cancer (TNBC) patients carry inherited mutations in the *BRCA1/2* breast cancer (BC) susceptibility genes. These mutations increase the risk of BC. However, the contribution of other BC susceptibility genes has not yet been determined. To this end, we aimed to investigate the prevalence of mutations in *BRCA1/2*-negative TNBC patients in Cyprus. Ninety-five cancer susceptibility genes were sequenced for 163 TNBC patients. The frequency of non-*BRCA* mutations and especially *PALB2* in TNBC patients in Cyprus appears to be higher compared to other populations, and half of the mutation-positive patients were diagnosed over the age of 60 years. Based on these results, we believe that *PALB2* and *TP53* along with *BRCA1/2* genetic testing could be beneficial for a large proportion of TNBC patients in Cyprus, irrespective of their age of diagnosis.

**Abstract:**

In Cyprus, approximately 9% of triple-negative (estrogen receptor-negative, progesterone receptor-negative, and human epidermal growth factor receptor 2-negative) breast cancer (TNBC) patients are positive for germline pathogenic variants (PVs) in *BRCA1/2*. However, the contribution of other genes has not yet been determined. To this end, we aimed to investigate the prevalence of germline PVs in *BRCA1/2*-negative TNBC patients in Cyprus, unselected for family history of cancer or age of diagnosis. A comprehensive 94-cancer-gene panel was implemented for 163 germline DNA samples, extracted from the peripheral blood of TNBC patients. Identified variants of uncertain clinical significance were evaluated, using extensive in silico investigation. Eight PVs (4.9%) were identified in two high-penetrance TNBC susceptibility genes. Of these, seven occurred in *PALB2* (87.5%) and one occurred in *TP53* (12.5%). Interestingly, 50% of the patients carrying PVs were diagnosed over the age of 60 years. The frequency of non-*BRCA* PVs (4.9%) and especially *PALB2* PVs (4.3%) in TNBC patients in Cyprus appears to be higher compared to other populations. Based on these results, we believe that *PALB2* and *TP53* along with *BRCA1/2* genetic testing could be beneficial for a large proportion of TNBC patients in Cyprus, irrespective of their age of diagnosis.

## 1. Introduction

Breast cancer (BC) is the most common cancer and the leading cause of cancer-related mortality in women worldwide [1]. Triple-negative breast cancer (TNBC) is a distinct, highly aggressive form of BC, characterized by lack of expression of the estrogen and progesterone receptors (ER/PR) as well as of human epidermal growth factor receptor 2 (HER2) in breast tumors [2]. It accounts for approximately 10–20% of all BC cases, and it occurs more frequently in young or premenopausal women and women of African American, Hispanic, and Ashkenazi Jewish ancestry [2,3,4]. Although TNBC lacks a specific therapeutic approach due to the absence of receptors for conventional targeted therapies, systemic cytotoxic chemotherapy can achieve good pathologic complete response (pCR) rates [5]. Survival rates are lower compared to other forms of breast cancer, with the majority of patients presenting with early recurrence and a high frequency of metastasis, especially to the central nervous system (20%) and viscera (29%) [3,6,7]. Accordingly, the five-year survival rate of patients with TNBC is estimated at 70% compared with >80% for all other breast cancer subtypes [8].

A high percentage of TNBC patients carry a germline pathogenic variant (PV) in one of the highly penetrant BC susceptibility genes, *BRCA1* and *BRCA2* [9]. Additionally, studies have also examined the contribution of other BC susceptibility genes including *ATM, BARD1, CHEK2*, *PALB2*, *PTEN*, *RAD51D*, and *TP53* to TNBC susceptibility [9,10,11,12,13,14]. Other cancer predisposition genes including *BRIP1*, *CDH1*, *CDKN2A*, *MLH1*, *MRE11A*, *MSH2*, *MSH6*, *NBN*, *NF1*, *PMS2*, *RAD50*, *RAD51C*, *FANCM, XRCC2,* and *MUTYH*—biallelic mutations—have also been investigated in TNBC [9,10,11,12,13,14,15]. Recently, it has been reported that 14.4% of TNBC patients carried PVs in established BC genes as well as other cancer susceptibility genes [9,14]. Of these, 6% harbored PVs in non-*BRCA* TNBC high-risk genes (odds ratio (OR) > 5.0) including *BARD1*, *PALB2*, and *RAD51D* and TNBC moderate-risk genes (OR > 2.0) including *BRIP1*, *RAD51C,* and *TP53* [9].

In a recent study from Cyprus, it has been reported that approximately 9% of TNBC patients were positive for germline PVs in the *BRCA1/2* genes [16]. However, the contribution of other BC susceptibility genes towards TNBC predisposition is not yet determined; thus, there is a great need to investigate the prevalence and distribution of germline PVs in other susceptibility genes.

In recent years, the expanding panel of BC susceptibility genes has necessitated the use of Next-Generation Sequencing (NGS). Consequently, NGS panel testing has been integrated into routine clinical practice. In this manuscript, we present the first NGS gene-panel investigation of TNBC patients in Cyprus who were negative for *BRCA1* and *BRCA2* germline PVs.

## 2. Results

### 2.1. Pathogenic Variants

Pathogenic variants were identified in two high-penetrance BC and TNBC susceptibility genes, in 8 out of 163 patients tested (4.9%) (Table 1). Of these, seven were detected in the *PALB2* gene (87.5%) and one was detected in the *TP53* gene (12.5%) (Table 1). Two *PALB2*-positive patients did not have a family history of breast, ovarian, pancreatic, or colorectal cancer, whereas two other patients had a strong family history (at least three first- or second-degree relatives diagnosed with breast, ovarian, or pancreatic cancer) (Table 1). It is noted that two *PALB2* PVs (c.487_488del and c.1685-2A>G) were present in 3 and 2 unrelated TNBC patients, respectively (Table 1). Besides family history, it is noteworthy that half of the PV carriers, (4 out of 8) were diagnosed with TNBC over the age of 60 years (Table 1) (*p*-value = 0.027, two-tailed *t*-test) (Figure S1).

### 2.2. Classification of Variants of Uncertain Clinical Significance (VUS)

In this study, 65 unique VUSs were identified and, of these, 21 were classified as deleterious by at least 75% of the in silico prediction tools (Table S1 and Figure S2). It is noted that five of the VUSs predicted as deleterious, are novel and have not been reported before in ClinVar, dbSNP, and LOVD databases or in any publications (Table S1). The American College of Medical Genetics and Genomics (ACMG) guidelines (Table S2) were followed for the classification of the 21 VUSs predicted as deleterious, according to which five out of the 21 variants were classified as likely benign, and 16 variants as VUS (Table S3).

**Figure 1:**
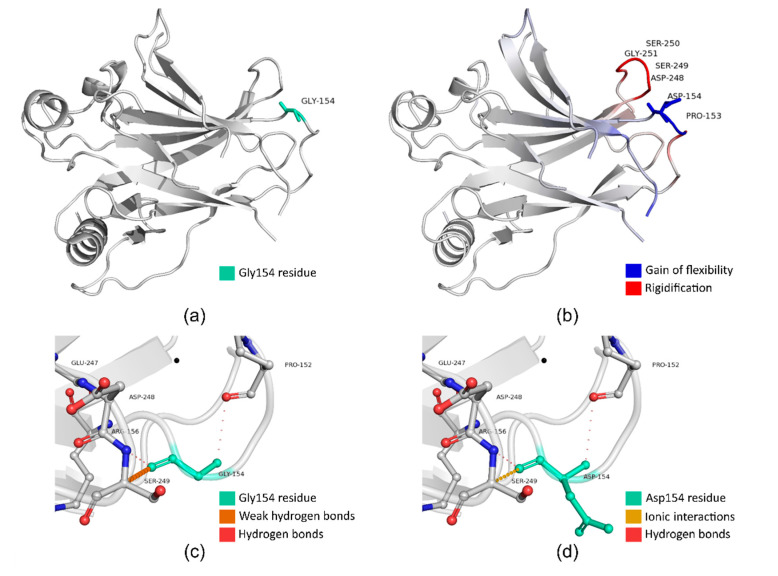
Structural and flexibility analysis of p.(Gly154Asp), a *TP53*-predicted deleterious Variants of Uncertain Clinical Significance (VUS): (**a**) schematic representation of the location of the mutated Gly154 amino acid on the wild-type TP53 protein (Protein Data Bank (PDB) ID: 1GZH); (**b**) visual representation of the differences in vibrational entropy energy upon mutagenesis; (**c**) atomic interactions of the wild-type (Gly154) amino acid; and (**d**) atomic interactions of the mutant (Asp154) amino acid. Analysis was performed using the DynaMut webserver (http://biosig.unimelb.edu.au/dynamut/).

Comprehensive in silico classification of the structural, functional, stability, and flexibility impact of the 21 VUSs predicted as deleterious was carried out (Tables S4 and S5). Despite its classification as a VUS, the missense *TP53* p.(Gly154Asp) variant is of particular interest (Figure 1) (ClinGen *TP53* Expert Panel Specifications to the ACMG/AMP variant interpretation guidelines) [17], since there is extensive evidence that supports its pathogenicity (Table S3). At the protein level, the Gly154 residue (Figure 1a) resides in the p53 DNA-binding domain (PF00870, amino acids 102-292), in which a large proportion of missense variants are classified as pathogenic (29%, 198 out of 674). Additionally, the Gly154 residue constitutes a mutational hotspot, since 25 somatic occurrences have been reported in the cancerhotspots.org database [18,19]. This variant is predicted to destabilize the protein structure and to disrupt the proper function of the TP53 protein, as it probably negatively affects the secondary structure of the protein and subsequently DNA binding (Table S4). As predicted, the phi (φ)/psi (ψ) angles are in the favored region for the wild-type Gly154 residue but in the outlier region for the mutant Asp154 residue. Additionally, the substitution causes a predicted decrease in molecule flexibility (−0.028 kcal.mol^−1^K^−1^) (Figure 1b). The wild-type amino acid may participate in intermolecular interactions (hydrogen bonds) with surrounding amino acids (Figure 1c), which would be probably hampered and shifted upon mutagenesis (Figure 1d). However, functional studies are needed for the accurate classification of this variant as well as all the other VUSs predicted as deleterious.

## 3. Discussion

This study is the first gene-panel NGS investigation of patients with TNBC in Cyprus who are negative for PVs in the *BRCA1* and *BRCA2* genes. The results present for the first time the prevalence of germline PVs in BC and other cancer predisposition genes in patients diagnosed with TNBC in Cyprus.

Among the 163 *BRCA1/2*-negative TNBC patients tested, 4.9% were positive for PVs in the ΤNBC susceptibility genes *PALB2* and *TP53*. This frequency is higher compared to a large multiethnic study of 10,901 TNBC patients, which demonstrated that only 3.1% of the *BRCA1/2*-negative study participants carried PVs in high-risk (*BARD1*, *PALB2*, and *RAD51D*) and moderate-risk (*TP53*, *BRIP1*, and *RAD51C*) TNBC susceptibility genes [9]. Similar observations are made when comparing the frequency of PVs reported here to that reported in the study of González-Rivera et al. in which 3.3% of the *BRCA1/2*-negative TNBC patients carried PVs in the high-penetrance TNBC susceptibility genes *BARD1* and *RAD51D* [20]. A large-scale study evaluated the performance of gene-panel testing in 35,409 women with a single diagnosis of BC. It has been reported that 3.3% of *BRCA1/2*-negative TNBC patients carried a PV in high- (*BARD1*, *PALB2*, and *RAD51D*) or moderate-risk (*TP53*, *BRIP1*, and *RAD51C*) TNBC susceptibility genes [10]. The frequency of PVs in TNBC susceptibility genes in Cyprus is about three times higher than the reported frequency in Chinese *BRCA*-negative TNBC patients, where only 1.36% were positive for PVs in the high-penetrance TNBC susceptibility genes *PALB2*, *RAD51D,* and *BARD1* [21]. The frequency of PVs reported here is also different compared to the frequency of PVs (1%) in TNBC susceptibility genes (*BARD1*, *PALB2*, *RAD51D*, *BRIP1*, *RAD51C,* and *TP53*) reported in a recently published study of the Georgia and California Cancer Registries [22]. In contrast, our results are similar to the frequency of PVs in TNBC susceptibility genes (4.8%) reported in Greek patients with TNBC [23]. However, the patients included in the Greek study were highly selected, having a strong family history (at least three breast, ovarian, or pancreatic cancer patients in the same family) and/or diagnosed earlier than the age of 35 years. This could in part explain the relatively high frequency of PVs reported in Greek samples. Nevertheless, this was not the case for our study cohort, where TNBC patients were unselected for age of onset and family history.

It is estimated that VUSs account for around 40% of the total number of variants identified in extended gene-panel sequencing studies, and their classification remains a major challenge [24]. In this study, nineteen patients (11.7%) carried VUS predicted as deleterious in established BC and other cancer susceptibility genes, while they did not carry any PV in a gene with confirmed BC association. In order to decipher further which VUS may be actionable, a detailed in silico investigation was carried out. The *TP53* VUS p.(Gly154Asp) identified in a patient with a family history of breast cancer is of particular interest. The variant resides in the p53 DNA binding domain, where 90% of all known *TP53* pathogenic variants are located [25] and is predicted to alter the DNA binding capacity of the p53 protein. Based on in silico prediction tools, it is likely that this missense variant is pathogenic, but functional studies and/or co-segregation or case-control studies are needed for the accurate classification of this and other VUS in TNBC patients.

The average age at initial TNBC diagnosis of our patient cohort is 50.55 ± 10.35, with a range from 27 to 75 years. This is similar to the average age of onset of the 10,901 TNBC patients recruited by the TNBC Consortium (TNBCC) and the Ambry Genetics clinical testing laboratory (49.9 ± 11.3 years) [9]. Among the 10,901 patients, 17.7% were diagnosed with TNBC over the age of 60 years, which is very similar to our population (17.8%) [9]. An interesting observation is that, among 29 study participants diagnosed over the age of 60 years, 13.8% were found positive for PVs in established BC. In contrast, in the large-scale multiethnic study of TNBC patients, 10.2% of patients diagnosed over the age of 60 years tested positive for germline PVs in established BC and other cancer susceptibility genes [9]. Considering that only half of the TNBC PV carriers fulfilled the NCCN guidelines, our results support the notion that all TNBC patients in Cyprus should be referred for genetic testing, irrespective of their age at diagnosis.

According to Cybulski et al., it is estimated that 34% of BC patients with a germline *PALB2* PV have a TNBC subtype [26]. *PALB2* PVs were the most prevalent in our cohort of *BRCA1/2*-negative TNBC samples (4.3%). Two large multiethnic studies of the TNBCC in collaboration with Ambry Genetics clinical testing laboratory and Myriad Genetics laboratory reported lower frequencies of *PALB2* PVs amongst TNBC patients (1.3% and 1.4%, respectively) [9,10]. A possible founder effect for two of the *PALB2* PVs identified (c.487_488del and c.1685-2A>G) in our study could partly explain the higher prevalence of *PALB2* PVs in our TNBC population. However, additional studies are required to draw more definite conclusions. The absence of a *CHEK2* PV in our TNBC cohort agrees with the reported association of *CHEK2* PVs with ER-positive BC and the absence of association with TNBC [9].

It is well known that *BRCA1/2* carriers with TNBC can benefit from targeted therapeutic strategies. Poly (ADP-ribose) polymerase (PARP) inhibitors (PARPi) can be used as a targeted treatment for germline *BRCA1/2*-positive BC patients with HER2-negative metastatic breast tumors [27]. Tumors harboring inherited or somatic pathogenic variants in other non-*BRCA* genes implicated in DNA repair pathways may also respond with a *BRCA*-like behavior to this particular class of drugs. An ongoing phase II clinical trial is investigating the antitumor efficacy of PARPi in patients with advanced HER2-negative or TNBC and a somatic or germline PV in a non-*BRCA1/2* Homologous Recombination pathway gene [28]. It is anticipated that, in the near future, patients harboring PVs in genes other than *BRCA1/2* may also benefit from these and other targeted therapies.

## 4. Materials and Methods

### 4.1. Study Population

This study involved patients with unilateral TNBC, referred to the Clinical Genetics Department of the Cyprus Institute of Neurology and Genetics for genetic counseling from 2004 to 2019. According to NCCN guidelines (version 1.2020), genetic testing should be offered to patients diagnosed with TNBC at age ≤60 years. The testing criteria were slightly modified from the international guidelines to reflect the situation in Cyprus. TNBC patients were referred for genetic counseling and subsequently underwent genetic testing irrespective of their age of disease onset. This study includes 163 TNBC patients who tested negative for germline PVs (including large genomic rearrangements) in the *BRCA1* and *BRCA2* genes and were unselected for family history of breast and/or ovarian cancer. Each study participant signed an informed consent form and agreed to undergo genetic testing.

The characteristics of the TNBC patients included in the study are summarized in Table 2.

### 4.2. Multigene Panel-Based Mutation Analysis

DNA samples from TNBC patients that tested negative for germline PVs in the *BRCA1* and *BRCA2* genes were analyzed further by targeted NGS, using a panel of 94 cancer susceptibility genes (Illumina TruSight Cancer Sequencing panel: #FC-121-0202). The panel contains oligos targeting and enriches more than 1700 exons, including coding regions and noncoding exon-flanking regions spanning the 94 cancer susceptibility genes (Table S6). The TruSight Rapid Capture kit was used for the preparation of the NGS library according to the manufacturer’s protocol (Illumina, #FC-140-1106). Sequencing was performed on the NextSeq 500 or the MiSeq Sequencing Platforms (Illumina, San Diego, CA, USA) with 75-bp or 150-bp paired-end reads. Screening for large rearrangements was not performed.

### 4.3. BARD1 Mutation Screening

Since the established high-risk TNBC susceptibility gene *BARD1* is not included in the TruSight Cancer Sequencing panel, *BARD1* mutation screening was performed for all samples included in this study by Sanger sequencing. All the coding exons and proximal splice site regions of the *BARD1* gene (LRG_297) were sequenced as described previously [29].

### 4.4. Bioinformatics Analysis

Sequence reads (2 × 150 bp or 2 × 75 bp) were aligned to the reference GRCh37 (hg19) human genome assembly using the Burrows-Wheeler Aligner—Maximal Exact Match (BWA-MEM) algorithm. Duplicates were detected by Picard (http://broadinstitute.github.io/picard/), and bases were recalibrated using the GATK suite and according to the best practice guidelines (https://www.broadinstitute.org/gatk). Variants were called with the GATK HaplotypeCaller and GenotypeGVCF, filtered following the GATK guidelines and functionally annotated using ANNOVAR (https://doc-openbio.readthedocs.io/projects/annovar/en/latest/).

### 4.5. Variant Selection

Multiple filtering criteria were used to identify PVs and variants of uncertain clinical significance (VUS). Variants with read depth of less than 30 were excluded from further analysis. Rare variants with minor allele frequency (MAF)—taken from the Exome Aggregation Consortium (ExAC) (https://gnomad.broadinstitute.org/)—less than or equal to 1% were selected for further investigation. Variation databases (ClinVar, https://www.ncbi.nlm.nih.gov/clinvar/; LOVD, http://www.lovd.nl/3.0/home; and dbSNP, https://www.ncbi.nlm.nih.gov/projects/SNP/) were explored to detect already published variants and their structural/functional effects. Only variants having a damaging effect on the protein sequence or structure—frameshift indels, splicing variants (±3 positions), nonsense, and missense variants reported as pathogenic or likely pathogenic in the literature—were considered as pathogenic. The regSNP-splicing tool (http://regsnps-splicing.ccbb.iupui.edu/) was used to prioritize synonymous variants based on their proximity to splice donor or acceptor sites. Alternative splicing events were tested by MaxEntScan (http://hollywood.mit.edu/burgelab/maxent/), BDGP: Splice Site Prediction by Neural Network (http://www.fruitfly.org/seq_tools/splice.html), Human Splicing Finder (HSF3.0) (http://www.umd.be/HSF3/), and NetGene2 (http://www.cbs.dtu.dk/services/NetGene2/). All PVs were confirmed by Sanger Sequencing as described elsewhere [29].

### 4.6. Classification of Variants of Uncertain Clinical Significance (VUS)

Further analysis of VUS was performed only for variants reported in established BC susceptibility genes (*BRCA1, BRCA2, PALB2, BARD1, RAD51D, ATM, CHEK2, PTEN,* and *TP53)* and other cancer susceptibility genes (*CDH1, BRIP1, CDKN2A, MLH1, MSH2, MSH6, NBN, NF1, PMS2, RAD51C, XRCC2, STK11, FANCM*, and *MUTYH*-biallelic). Protein features such as amino acid conservation among species, wild-type/mutant amino acid biophysical and/or biochemical properties, amino acid localization within the protein, and potential impact on mRNA were investigated using several in silico prediction tools [30,31,32,33,34,35,36,37,38,39]. Variants considered as deleterious were those classified as such by at least 75% of the prediction tools. The American College of Medical Genetics and Genomics (ACMG) standards and guidelines for the interpretation of sequence variants were followed for variant classification [40]. In addition, a comprehensive in silico classification scheme for the interpretation of the stability [41,42,43,44,45,46,47,48,49,50,51,52,53,54], structural [55,56,57,58], functional [55,56,57,58], and flexibility [51] impact of VUS predicted as deleterious was carried out, using experimentally solved or in silico predicted protein structures [59,60]. The detailed workflow followed for the in silico classification of VUS is described in the Supplementary Methods. All VUSs predicted as deleterious were confirmed by Sanger Sequencing as described elsewhere [29].

### 4.7. Statistical Analysis

The R (v3.3.2) (https://www.r-project.org/) statistical computing language was used for statistical analysis. The Shapiro–Wilk’s test was used to test whether the underlying distribution was normal. Carriers and non-carriers of PVs were compared for numerical variables with independent samples two-tailed t-test (for normally distributed values). Two-tailed *p*-values lower than 0.05 were considered to be statistically significant.

## 5. Conclusions

In conclusion, our study identified eight PVs in ΤΝΒC susceptibility genes in eight unrelated *BRCA1/2*-negative TNBC patients in Cyprus (4.9%), unselected for family history and age at disease diagnosis. Although our sample size is small, the reported frequency is higher compared to other populations and may have important clinical implications. Additionally, the frequency of *PALB2* PVs (4.3%) appears to be higher compared to other populations (1.3–1.4%), while half of the PV-positive patients were diagnosed over the age of 60 years. We suggest that *PALB2* and *TP53* along with *BRCA1/2* genetic testing could be beneficial for a substantial proportion of TNBC patients in Cyprus, irrespective of their age at diagnosis.

## Figures and Tables

**Table 1:** Summary of the detected pathogenic variants and patient characteristics.

Gene	Exon	cDNA Change	Amino Acid Change	FH	^†^ Age	^‡^ Frequency Per PV (%)	^§^ Frequency Per Gene (%)
*PALB2*	4	c.487_488del	p.(Val163Leufs*4)	2 × BC	51	3/8 (37.5%)	7/8 (87.5%)
*PALB2*	4	c.487_488del	p.(Val163Leufs*4)	4 × BC	65		
*PALB2*	4	c.487_488del	p.(Val163Leufs*4)	-	67		
*PALB2*	5	c.1685-2A>G	p.?	7 × BC	54	2/8 (25%)	
*PALB2*	5	c.1685-2A>G	p.?	-	48		
*PALB2*	12	c.3350+4A>G	p.?	BC	50	1/8 (12.5%)	
*PALB2*	3	c.172_175del	p.(Gln60Argfs*7)	BC	67	1/8 (12.5%)	
*TP53*	6	c.584T>C	p.(Ile195Thr)	OC	68	1/8 (12.5%)	1/8 (12.5%)

FH: Family history of cancer, with first- or second-degree relatives diagnosed with breast, ovarian, pancreatic, or colorectal cancer. ^†^ Age at diagnosis with Triple-Negative Breast Cancer (years). ^‡^ Patients carrying the pathogenic variant (PV)/patients tested positive for PVs in established breast cancer (BC) susceptibility genes. ^§^ Patients carrying PVs per gene/patients tested positive for PVs in established BC susceptibility genes. Mutation nomenclature according to LRG_308t1 (*PALB2*) and LRG_321t1 (*TP53*). BC, breast cancer; OC, ovarian cancer; PV, pathogenic variant.

**Table 2:** Study population characteristics.

Characteristics	No. of Patients (%)	^†^ PV Carriers (%)
Total female patients	163 (100%)	8 (4.9%)
Age at diagnosis, y		
Mean ± SD (range)	50.6 ± 10.4 (27–75)	58.8 ± 8.8 (48–68)
<20	0	0
20–29	3 (1.8%)	0
30–39	22 (13.5%)	0
40–49	52 (31.9%)	1 (12.5%)
50–59	48 (29.4%)	3 (37.5%)
≥60	38 (23.3%)	4 (50%)
* Family history of cancer		
Breast or ovarian cancer	83 (50.9%)	6 (75%)
Pancreatic or colorectal cancer (No BC or OC)	17 (10.4%)	0
No breast, ovarian, pancreatic or colorectal	63 (38.7%)	2 (25%)

* First- or second-degree relatives ^†^ PVs, Pathogenic variants (frameshift; splice site variants, ±3 positions; nonsense; missense variants reported as pathogenic or likely pathogenic) reported in established breast cancer susceptibility genes (*PALB2* and *TP53*). BC, breast cancer; OC, ovarian cancer.

## Supplementary Materials

The following are available online at https://www.mdpi.com/2072-6694/12/11/3140/s1, Supplementary Methods: Classification of variants with uncertain clinical significance, Figure S1: Plots demonstrating the distribution of age at TNBC diagnosis of *BRCA1/2*-negative women in Cyprus, Table S1: Summary of the predicted deleterious VUSs in established BC and other cancer susceptibility genes, Figure S2: Box plot and histogram demonstrating the distribution of pathogenic variants and in silico predicted deleterious VUSs in established BC and other cancer susceptibility genes among all study samples, Table S2: Criteria as specified by the American College of Medical Genetics (ACMG) standards and guidelines for the interpretation of sequence variants, Table S3: Classification of the in silico predicted deleterious VUS based on the ACMG standards and guidelines for interpretation of sequence variants, Table S4: In silico assessment of the structural, functional, stability, and flexibility impact of the predicted deleterious VUSs in BC susceptibility genes, Table S5: In silico assessment of the structural, functional, stability, and flexibility impact of the predicted deleterious VUSs in other cancer susceptibility genes, Table S6: TruSight Cancer (Illumina) target genes in alphabetical order.
