# Supplementary material for: NGS Panel Testing of Triple-Negative Breast Cancer Patients in Cyprus: A Study of BRCA-Negative Cases
Source: Cancers (Basel). 2020 Oct 27;12(11):3140. doi: 10.3390/cancers12113140 (PMC7692082; doi:10.3390/cancers12113140)
Supplement: Supplementary file 1 [file cancers-12-03140-s001.pdf]

Article

NGS Panel Testing of Triple-Negative Breast Cancer Patients in Cyprus; a Study of *BRCA*-Negative Cases

Maria Zanti [1,2,3], Maria A. Loizidou [1,2], Kyriaki Michailidou [2,4], Panagiota Pirpa [1], Christina Machattou [1], Yiola Marcou [5], Flora Kyriakou [5], Eleni Kakouri [5], George A. Tanteles [2,6], Elena Spanou [6], George M. Spyrou [2,3], Kyriacos Kyriacou [1,2] and Andreas Hadjisavvas [1,2,*]

1. Department of Electron Microscopy/Molecular Pathology, The Cyprus Institute of Neurology and Genetics, 6 Iroon Avenue, 2371 Agios Dhometios, Nicosia, Cyprus; mariaz@cing.ac.cy (M.Z.); loizidou@cing.ac.cy (M.A.L.); panagiotap@cing.ac.cy (P.P.); christinam@cing.ac.cy (C.M.); kyriacos@cing.ac.cy (K.K.)
2. Cyprus School of Molecular Medicine, The Cyprus Institute of Neurology and Genetics, 6 Iroon Avenue, 2371 Agios Dhometios, Nicosia, Cyprus; kyriakimi@cing.ac.cy (K.M.); gtanteles@cing.ac.cy (G.A.T.); georges@cing.ac.cy (G.M.S.)
3. Bioinformatics Department, The Cyprus Institute of Neurology and Genetics, 6 Iroon Avenue, 2371 Agios Dhometios, Nicosia, Cyprus
4. Biostatistics Unit, The Cyprus Institute of Neurology and Genetics, 6 Iroon Avenue, 2371 Agios Dhometios, Nicosia, Cyprus
5. Department of Medical Oncology, Bank of Cyprus Oncology Center, 32 Acropoleos Avenue, 2012 Strovolos, Nicosia, Cyprus; yiola.marcou@bococ.org.cy (Y.M.); florakyriakou@gmail.com (F.K.); eleni.kakouri@bococ.org.cy (E.K.)
6. Clinical Genetics Department, The Cyprus Institute of Neurology and Genetics, 6 Iroon Avenue, 2371 Agios Dhometios, Nicosia, Cyprus; elenaa@cing.ac.cy

* Correspondence: ahsavvas@cing.ac.cy; Tel.: +357-22392739

Supplementary Methods

Classification of variants with uncertain clinical significance

The pathogenicity of variants with uncertain clinical significance (VUS) was at first, investigated using nine in silico prediction tools namely: SIFT [1], PolyPhen2 [2], LRT [3], MutationAssessor [4] PROVEAN [5], MutationTaster2 [6], CADD [7], Align-GVGD [8], UMD-predictor [9], and BayesDel [10]. These tools combine features such as amino acid conservation among species, wild-type/mutant amino acid biophysical and/or biochemical properties, amino acid localization within the protein and potential impact on mRNA. Variants considered as deleterious were those classified as such by at least 75% of the prediction tools. The American College of Medical Genetics and Genomics (ACMG) standards and guidelines for the interpretation of sequence variants (Table S3), were followed for variant classification [11]. The ClinGen *TP53* Expert Panel Specifications to the ACMG/AMP Variant Interpretation Guidelines (version 1) were used for the interpretation of *TP53* variants [12].

Experimentally derived three-dimensional protein structures were obtained from the Protein Data Bank (PDB) (https://www.wwpdb.org/). Comparative structural modelling was carried out for unknown protein structures using the template-based web server, I-TASSER (https://zhanglab.ccmb.med.umich.edu/I-TASSER/). The accuracy of the method was assessed based on the I-TASSER C-score, a confidence score for the quality of the predicted models. The C-score was calculated based on the significance of threading template alignments and the convergence parameters of the structure assembly simulations. It typically ranges between -5 and 2, and a value higher than -1.5 signifies a high accuracy of the predicted model [13]. I-TASSER was selected for protein structure modeling, since it outperformed other servers according to results from the 13th Community Wide Experiment on the Critical Assessment of Techniques for Protein Structure Prediction (CASP13) [14]. Protein structures were fixed and global energy (ΔG) was lowered when necessary, using the FoldX4.0 suite (http://foldxsuite.crg.eu/). The PyMOL software (https://pymol.org/2/) was used for the visualization of the protein molecules.

A comprehensive in silico classification scheme for the interpretation of the stability, structural, functional, and flexibility impact of VUS predicted as deleterious, was carried out. The effect of each point substitution on protein stability was predicted and computed (FoldX [15], I-Mutant3.0 [16], MUpro [17], CUPSAT [18], PoPMuSiC [19], DynaMut [20], ENCoM [21], DUET [22], SDM [23] and mCSM-Stability [24]), as the difference in folding free energy ($\Delta\Delta G$), between the wild-type and mutant protein compounds.

Studies have shown that pathogenic variants (PVs) reducing the protein stability by > 2 kcal/mol -which roughly corresponds to the energy of two hydrogen bonds for proteins in solution- are severely debilitating and disrupt the proper function of the protein [25–27]. We followed the classification system supported by Seifi and Walter [28], according to which, variants were classified as stabilizing ($\Delta\Delta G > 1.5$), neutral ($-1.5 < \Delta\Delta G < 1.5$) and destabilizing ($\Delta\Delta G$

< -1.5). The mCSM-Protein-Protein and mCSM-Protein-DNA webservers, were used to calculate the effect of substitutions on protein-protein and protein-DNA complex affinities.

The SAAPdap/SAAPpred [29], missense3D [30], SuSPect [31], and SNAP2 webservers [32], were used to estimate the effect of amino acid substitutions on protein structure and function, by combining protein structural features. Finally, the DynaMut webserver [20], was used for protein flexibility analysis. The vibrational entropy energy ($\Delta\Delta SVib$) upon mutagenesis, contributes significantly to the binding free energies of molecules. DynaMut implements ENCoM [21], to calculate $\Delta\Delta SVib$ as the difference between the vibrational energy of wild-type and mutant structures.

References

1. Vaser, R.; Adusumalli, S.; Ngak Leng, S.; Sikic, M.; Ng, P.C. SIFT missense predictions for genomes. *Nat. Protoc.* **2015**, *11*, doi:10.1038/nprot.2015.123.
2. Adzhubei, I.A.; Schmidt, S.; Peshkin, L.; Ramensky, V.E.; Gerasimova, A.; Bork, P.; Kondrashov, A.S.; Sunyaev, S.R. *A method and server for predicting damaging missense mutations*; 2010; Vol. 7, pp. 248–249;.
3. Chun, S.; Fay, J.C. Identification of deleterious mutations within three human genomes. *Genome Res.* **2009**, *19*, 1553–61, doi:10.1101/gr.092619.109.
4. Reva, B.; Antipin, Y.; Sander, C. Predicting the functional impact of protein mutations: application to cancer genomics. *Nucleic Acids Res* **2011**, *39*, 118, doi:10.1093/nar/gkr407.
5. Choi, Y.; Sims, G.E.; Murphy, S.; Miller, J.R.; Chan, A.P. Predicting the Functional Effect of Amino Acid Substitutions and Indels. *PLoS One* **2012**, *7*, e46688, doi:10.1371/journal.pone.0046688.
6. Schwarz, J.M.; Rödelsperger, C.; Schuelke, M.; Seelow, D. MutationTaster evaluates disease-causing potential of sequence alterations. *Nat. Methods* **2010**, *7*, 575–576, doi:10.1038/nmeth0810-575.
7. Kircher, M.; Witten, D.M.; Jain, P.; O'Roak, B.J.; Cooper, G.M.; Shendure, J. A general framework for estimating the relative pathogenicity of human genetic variants. *Nat. Genet.* **2014**, *46*, 310–315, doi:10.1038/ng.2892.
8. Tavtigian, S. V; Deffenbaugh, A.M.; Yin, L.; Judkins, T.; Scholl, T.; Samollow, P.B.; de Silva, D.; Zharkikh, A.; Thomas, A. Comprehensive statistical study of 452 BRCA1 missense substitutions with classification of eight recurrent substitutions as neutral. *J. Med. Genet.* **2006**, *43*, 295–305, doi:10.1136/jmg.2005.033878.
9. Salgado, D.; Desvignes, J.-P.; Rai, G.; Blanchard, A.; Miltgen, M.; Pinard, A.; Lévy, N.; Collod-Béroud, G.; Béroud, C. UMD-Predictor: A High-Throughput Sequencing Compliant System for Pathogenicity Prediction of any Human cDNA Substitution. *Hum. Mutat.* **2016**, *37*, 439–446, doi:10.1002/humu.22965.
10. Feng, B.-J. PERCH: A Unified Framework for Disease Gene Prioritization. *Hum. Mutat.* **2017**, *38*, 243–251, doi:10.1002/humu.23158.
11. Richards, S.; Aziz, N.; Bale, S.; Bick, D.; Das, S.; Gastier-Foster, J.; Grody, W.W.; Hegde, M.; Lyon, E.; Spector, E.; et al. Standards and guidelines for the interpretation of sequence variants: a joint consensus recommendation of the American College of Medical Genetics and Genomics and the Association for Molecular Pathology. *Genet Med* **2015**, *17*, 405–423, doi:10.1038/gim.2015.30.
12. TP53 Variant Curation Expert Panel *ClinGen TP53 Expert Panel Specifications to the ACMG/AMP Variant Interpretation Guidelines Version 1*; 2019;
13. Yang, J.; Zhang, Y. Protein Structure and Function Prediction Using I-TASSER. *Curr. Protoc. Bioinforma.* **2015**, *52*, 5.8.1-5.8.15, doi:10.1002/0471250953.bi0508s52.
14. Guzenko, D.; Lafita, A.; Monastyrskyy, B.; Kryshtafovych, A.; Duarte, J.M. Assessment of protein assembly prediction in CASP13. *Proteins* **2019**, doi:10.1002/prot.25795.
15. Schymkowitz, J.; Borg, J.; Stricher, F.; Nys, R.; Rousseau, F.; Serrano, L. The FoldX web server: an online force field., doi:10.1093/nar/gki387.
16. Capriotti, E.; Fariselli, P.; Casadio, R. I-Mutant2.0: predicting stability changes upon mutation from the protein sequence or structure. *Nucleic Acids Res.* **2005**, *33*, W306-10, doi:10.1093/nar/gki375.
17. Cheng, J.; Randall, A.; Baldi, P. Prediction of Protein Stability Changes for Single-Site Mutations Using Support Vector Machines. *Proteins* **2006**, *62*, 1125–1132, doi:10.1002/prot.20810.
18. Parthiban, V.; Gromiha, M.M.; Schomburg, D. CUPSAT: Prediction of protein stability upon point mutations. *Nucleic Acids Res.* **2006**, *34*, 239–242, doi:10.1093/nar/gkl190.
19. Dehouck, Y.; Kwasigroch, J.M.; Gilis, D.; Rooman, M. PoPMuSiC 2.1: A web server for the estimation of protein stability changes upon mutation and sequence optimality. *BMC Bioinformatics* **2011**, *12*, 151, doi:10.1186/1471-2105-12-151.
20. Rodrigues, C.H.; Pires, D.E.; Ascher, D.B.; RenéRen, I.; Rachou, R.; Oswaldo Cruz, F. DynaMut: predicting the impact of mutations on protein conformation, flexibility and stability. *Nucleic Acids Res.* **2018**, *46*, doi:10.1093/nar/gky300.
21. Frappier, V.; Chartier, M.; Najmanovich, R.J. ENCoM server: exploring protein conformational space and the effect of mutations on protein function and stability. *Nucleic Acids Res.* **2015**, *43*, 395–400, doi:10.1093/nar/gkv343.
22. Pires, D.E. V.; Ascher, D.B.; Blundell, T.L. DUET: a server for predicting effects of mutations on protein stability using an integrated computational approach. *Nucleic Acids Res.* **2014**, *42*, W314–W319, doi:10.1093/nar/gku411.
23. Pandurangan, A.P.; Ochoa-Montaño, B.; Ascher, D.B.; Blundell, T.L. SDM: a server for predicting effects of mutations on protein stability. *Nucleic Acids Res.* **2017**, *45*, W229–W235, doi:10.1093/nar/gkx439.
24. Pires, D.E. V.; Ascher, D.B.; Blundell, T.L. mCSM: predicting the effects of mutations in proteins using graph-based signatures. *Bioinformatics* **2014**, *30*, 335–342, doi:10.1093/bioinformatics/btt691.
25. Lindberg, M.J.; Bystrom, R.; Boknas, N.; Andersen, P.M.; Oliveberg, M. Systematically perturbed folding patterns of

amyotrophic lateral sclerosis (ALS)-associated SOD1 mutants. *Proc. Natl. Acad. Sci.* **2005**, *102*, 9754–9759, doi:10.1073/pnas.0501957102.
26. Randles, L.G.; Lappalainen, I.; Fowler, S.B.; Moore, B.; Hamill, S.J.; Clarke, J. Using Model Proteins to Quantify the Effects of Pathogenic Mutations in Ig-like Proteins. *J. Biol. Chem.* **2006**, *281*, 24216–24226, doi:10.1074/jbc.M603593200.
27. Sheu, S.Y.; Yang, D.Y.; Selzle, H.L.; Schlag, E.W. Energetics of hydrogen bonds in peptides. *Proc. Natl. Acad. Sci. U. S. A.* **2003**, *100*, 12683–12687, doi:10.1073/pnas.2133366100.
28. Seifi, M.; Walter, M.A. Accurate prediction of functional, structural, and stability changes in PITX2 mutations using in silico bioinformatics algorithms. *PLoS One* **2018**, *13*, e0195971, doi:10.1371/journal.pone.0195971.
29. Al-Numair, N.S.; Martin, A.C.R. The SAAP pipeline and database: tools to analyze the impact and predict the pathogenicity of mutations. *BMC Genomics* **2013**, *14 Suppl 3*, S4, doi:10.1186/1471-2164-14-s3-s4.
30. Ittisoponpisan, S.; Islam, S.A.; Khanna, T.; Alhuzimi, E.; David, A.; Sternberg, M.J.E. Can Predicted Protein 3D Structures Provide Reliable Insights into whether Missense Variants Are Disease Associated? *J. Mol. Biol.* **2019**, *431*, 2197–2212, doi:10.1016/j.jmb.2019.04.009.
31. Yates, C.M.; Filippis, I.; Kelley, L.A.; Sternberg, M.J.E. SuSPect: Enhanced prediction of single amino acid variant (SAV) phenotype using network features. *J. Mol. Biol.* **2014**, *426*, 2692–2701, doi:10.1016/j.jmb.2014.04.026.
32. Bromberg, Y.; Rost, B. SNAP: predict effect of non-synonymous polymorphisms on function. *Nucleic Acids Res.* **2007**, *35*, 3823–3835, doi:10.1093/nar/gkm238.

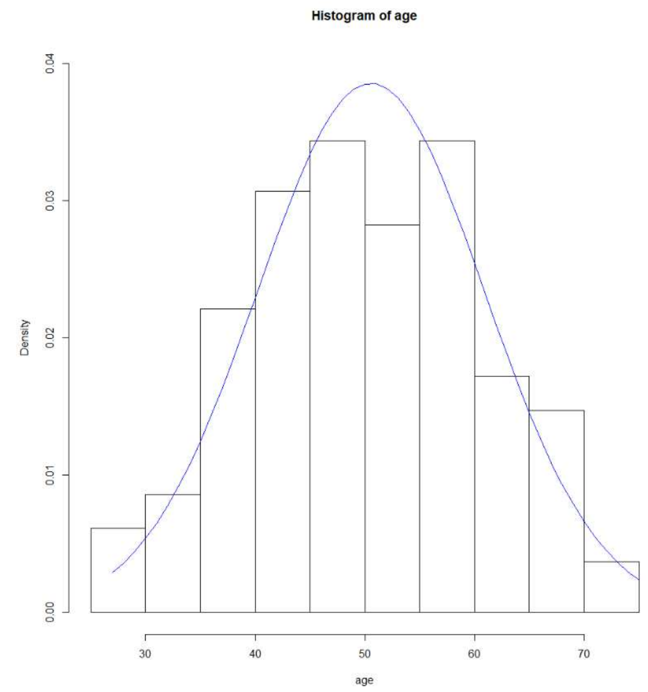

(a)

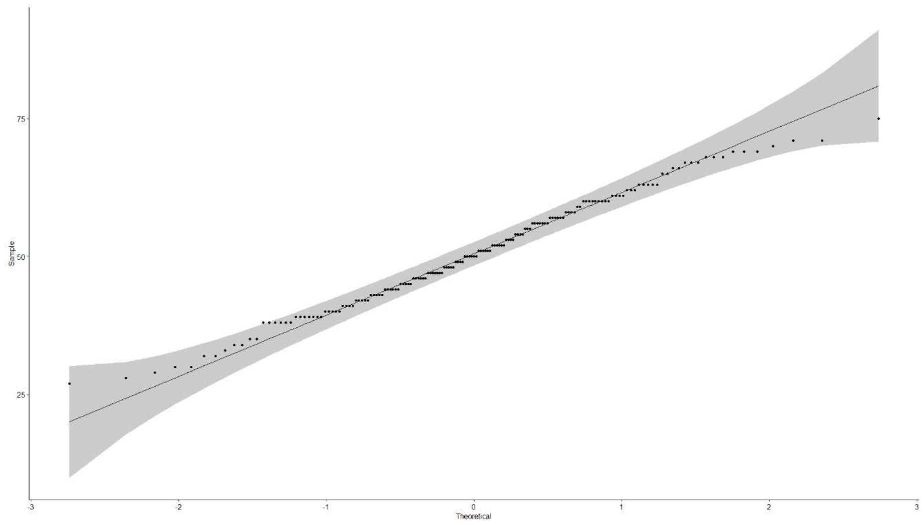

(b)

Figure S1. Plots demonstrating the distribution of age at TNBC diagnosis of *BRCA1*/2-negative women in Cyprus. (a) Distribution of age at TNBC diagnosis of *BRCA1*/2-negative women in Cyprus. Mean Age = 50.55, Standard Deviation = 10.35; (b) Quantile - Quantile (Q-Q) plot showing the correlation and normal distribution among age at TNBC diagnosis of *BRCA1*/2-negative women in Cyprus. 45-degree reference line is also plotted, W = 0.989072, p-value = 0.239018.

Table S1. Summary of the predicted deleterious VUS in established BC and other cancer susceptibility genes.

	Gene	Exon	cDNA change	Amino acid change	In silico pathogenicity prediction tools										In silico tools for the prediction of aberrant splicing			
					SIFT	PolyPhen2	LRT	Mutation Assessor	PROVEAN	Mutation Taster2	CADD	Align-GVGD	BayesDel	UMD-Predictor	MaxEntScan	BDGP	HSF 3.0	NetGene2
Established BC susceptibility genes	*PALB2*	13	c.3428T>A	p.(Leu1143His)	*0.004*	*0.995*	Neu	*1.955*	*-4.34*	*Del*	*24.8*	*C65*	-0.284	*84*	N/A	N/A	N/A	N/A
	BARD1	11	c.2189A>C	p.(Gln730Pro)	*0.010*	*1*	*Del*	*2.54*	*-2.5*	*Del*	*27.4*	*C65*	*0.374*	*93*	N/A	N/A	N/A	N/A
	BARD1	5	c.1319A>G	p.(Asp440Gly)	*0*	*0.97*	*Del*	*0.89*	*-5.5*	*Del*	*25*	*C65*	*0.344*	*78*	N/A	No aberration	*New ESS*	No aberration
	RAD51D	5	c.412A>C	p.(Asn138His)	0.120	*0.996*	*Del*	*2.35*	-2.11	*Del*	*23.7*	*C65*	-0.065	*90*	N/A	N/A	N/A	N/A
	RAD51D	**6**	**c.526T>C**	**p.(Phe176Leu)**	*0.010*	*0.97*	*Del*	*0.98*	*-4.4*	*Del*	*30*	*C65*	*0.191*	*66*	N/A	N/A	N/A	N/A
	ATM	60	c.8734A>G	p.(Arg2912Gly)	*0*	*1*	*Del*	*3.345*	*-5.83*	*Del*	*29.3*	*C65*	*0.198*	*100*	N/A	N/A	N/A	N/A
	ATM	22	c.3256C>T	p.(Arg1086Cys)	0.223	0.042	*Del*	*1.19*	*-3.63*	*Del*	*23.1*	*C65*	-0.144	*96*	N/A	N/A	N/A	N/A
	CHEK2	5	c.608A>G	p.(Asp203Gly)	*0.010*	*1*	*Del*	*3.32*	*-4.8*	*Del*	*29.9*	*C65*	*0.430*	*100*	N/A	N/A	N/A	N/A
	*TP53**	5	c.461G>A	p.(Gly154Asp)	N/A	N/A	*Del*	*2.19*	*-6.48*	*Del*	19.71	*C65*	*0.345*	*87*	N/A	N/A	N/A	N/A
Other cancer susceptibility genes	*CDH1*	2	c.160A>G	p.(Arg54Gly)	*0.044*	0.881	Neu	*2.615*	*-2.8*	*Del*	*25*	*C65*	-0.130	*96*	N/A	N/A	N/A	N/A
	BRIP1	7	c.797C>T	p.(Thr266Met)	*0.001*	*1*	*Del*	*3.655*	*-5.4*	*Del*	*27.3*	*C65*	0.217	*93*	N/A	N/A	N/A	N/A
	MSH2	1	c.182A>C	p.(Gln61Pro)	*0.037*	0.606	*Del*	*2.285*	-1.71	*Del*	*23.2*	*C65*	0.213	*81*	N/A	N/A	N/A	N/A
	MSH6	4	c.1729C>T	p.(Arg577Cys)	*0.040*	0.869	*Del*	*2.35*	-2.11	*Del*	*23.7*	*C65*	*0.293*	*90*	N/A	N/A	N/A	N/A
	NF1	**9**	**c.901G>A**	**p.(Asp301Asn)**	*0.006*	0.922	*Del*	*2.05*	*-3.2*	*Del*	*24.8*	C15	-0.120	*81*	N/A	N/A	N/A	N/A
	NF1	**40**	**c.5953C>T**	**p.(Leu1985Leu)**	N/A	N/A	N/A	N/A	N/A	N/A	N/A	N/A	N/A	N/A	N/A	N/A	*New ESS/Broken ESE*	N/A
	PMS2	12	c.2068A>C	p.(Lys690Gln)	0.137	*0.998*	*Del*	*2.11*	*-2.9*	*Del*	*24.3*	C45	*0.032*	*66*	N/A	N/A	N/A	N/A
	PMS2	12	c.2012C>T	p.(Thr671Met)	*0.028*	*0.979*	Neu	*2.2*	-1.85	*Del*	*27.8*	*C65*	-0.160	*75*	N/A	N/A	N/A	N/A
	RAD51C	5	c.790G>A	p.(Gly264Ser)	*0.010*	0.03	*Del*	*1.32*	*-2.6*	*Del*	*24.9*	*C55*	-0.274	50	N/A	N/A	N/A	N/A
	RAD51C	7	c.935G>A	p.(Arg312Gln)	*0*	*1*	*Del*	*3.48*	*-3.4*	*Del*	*34*	C35	*0.228*	*78*	N/A	N/A	N/A	N/A
	STK11	**8**	**c.1087A>C**	**p.(Thr363Pro)**	0.054	*0.978*	*Del*	*2.005*	-1.73	*Del*	*22.1*	C35	*0.138*	*100*	N/A	N/A	N/A	N/A
	FANCM	**21**	**c.5692G>A**	**p.(Val1898Met)**	*0*	*1*	*Del*	*2.485*	-2.22	*Del*	*28.3*	C15	*0.182*	*72*	N/A	N/A	N/A	N/A

BC, Breast cancer; Del, Deleterious; ESS, Exonic splicing silencer; ESE, Exonic splicing enhancer; Neu, Neutral; N/A, non-applicable; novel variants in **bold**; predicted deleterious scores in *italics*. Mutation nomenclature according to: *ATM* (LRG_135t1), *BARD1* (LRG_297t1), *BRIP1* (LRG_300t1), *CDH1* (LRG_301t1), *CHEK2* (LRG_302t1), *FANCM* (LRG_502t1), *MSH2* (LRG_218t1), *MSH6* (LRG_219t1), *NF1* (LRG_214), *PALB2* (LRG_308t1), *PMS2* (LRG_161t1), *RAD51C* (LRG_314t1), *RAD51D* (LRG_516t1), *STK11* (LRG_319t1), *TP53* (LRG_321t1). *According to ClinGen *TP53* Expert Panel Specifications to the ACMG/AMP Variant Interpretation Guidelines, SIFT and PolyPhen2 in silico modeling programs should not be used for *TP53* missense variants. Concordance of two predictors is recommended: Align-GVGD (Class C15 and higher) and BayesDel (scores > 0.16).

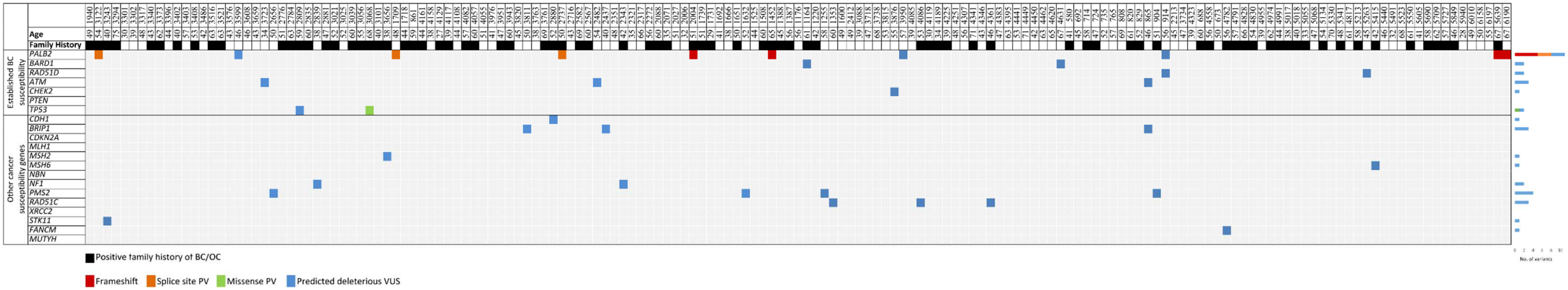

Figure S2. Box plot and histogram demonstrating the distribution of pathogenic variants and in silico predicted deleterious VUS in established BC and other cancer susceptibility genes, among all study samples. Patients with positive family history of breast or ovarian cancer in black, frameshift pathogenic variants in red, splice site pathogenic variants in orange, nonsense pathogenic variants in purple, missense pathogenic variants in green and in silico predicted deleterious VUS in blue. BC, breast cancer; OC, ovarian cancer; PV, pathogenic variant; VUS, variant of uncertain clinical significance.

Table S2. Criteria as specified by the American College of Medical Genetics (ACMG) standards and guidelines for the interpretation of sequence variants.

Pathogenic	Very Strong	PVS1	Null variant (nonsense, frameshift, canonical ±1 or 2 splice sites, initiation codon, single or multi-exon deletion) in a gene where LOF is a known mechanism of disease
	Strong	PS1	Same amino acid change as a previously established pathogenic variant regardless of nucleotide change
		PS2	*De novo (both maternity and paternity confirmed) in a patient with the disease and no family history*
		PS3	Well-established in vitro or in vivo functional studies supportive of a damaging effect on the gene or gene product
		PS4	The prevalence of the variant in affected individuals is significantly increased compared with the prevalence in controls
	Moderate	PM1	Located in a mutational hot spot and/or critical and well-established functional domain (e.g., active site of an enzyme) without benign variation
		PM2	Absent from controls (or at extremely low frequency if recessive) (Table 6) in Exome Sequencing Project, 1000 Genomes Project, or Exome Aggregation Consortium
		PM3	For recessive disorders, detected in trans with a pathogenic variant
		PM4	Protein length changes as a result of in-frame deletions/insertions in a non-repeat region or stop-loss variants
		PM5	Novel missense change at an amino acid residue where a different missense change determined to be pathogenic has been seen before
		PM6	Assumed de novo, but without confirmation of paternity and maternity
	Supporting	PP1	Co-segregation with disease in multiple affected family members in a gene definitively known to cause the disease
		PP2	Missense variant in a gene that has a low rate of benign missense variation and in which missense variants are a common mechanism of disease
		PP3	Multiple lines of computational evidence support a deleterious effect on the gene or gene product (conservation, evolutionary, splicing impact, etc.)
		PP4	Patient's phenotype or family history is highly specific for a disease with a single genetic etiology
		PP5	Reputable source recently reports variant as pathogenic, but the evidence is not available to the laboratory to perform an independent evaluation
Benign	Stand-alone	BA1	Allele frequency is >5% in Exome Sequencing Project, 1000 Genomes Project, or Exome Aggregation Consortium
	Strong	BS1	Allele frequency is greater than expected for disorder
		BS2	Observed in a healthy adult individual for a recessive (homozygous), dominant (heterozygous), or X-linked (hemizygous) disorder, with full penetrance expected at an early age
		BS3	Well-established in vitro or in vivo functional studies show no damaging effect on protein function or splicing
		BS4	Lack of segregation in affected members of a family
	Supporting	BP1	Missense variant in a gene for which primarily truncating variants are known to cause disease
		BP2	Observed in trans with a pathogenic variant for a fully penetrant dominant gene/disorder or observed in cis with a pathogenic variant in any inheritance pattern
		BP3	In-frame deletions/insertions in a repetitive region without a known function
		BP4	Multiple lines of computational evidence suggest no impact on gene or gene product (conservation, evolutionary, splicing impact, etc.)
		BP5	Variant found in a case with an alternate molecular basis for disease
		BP6	Reputable source recently reports variant as benign, but the evidence is not available to the laboratory to perform an independent evaluation
		BP7	A synonymous variant for which splicing prediction algorithms predict no impact to the splice consensus sequence nor the creation of a new splice site AND the nucleotide is not highly conserved

Table S3. Classification of the in silico predicted deleterious VUS based on the ACMG standards and guidelines for the interpretation of sequence variants.

	Gene	Exon	cDNA change	Amino acid change	PVS	PS1	PS2	PS3	PS4	PM1	PM2	PM3	PM4	PM5	PM6	PP1	PP2	PP3	PP4	PP5	BA1	BS1	BS2	BS3	BS4	BP1	BP2	BP3	BP4	BP5	BP6	BP7	Classification
Established BC susceptibility genes	*PALB2*	13	c.3428T>A	p.(Leu1143His)										✓				✓				✓				✓					✓		Likely Benign
	BARD1	11	c.2189A>C	p.(Gln730Pro)							✓							✓								✓							VUS
	BARD1	5	c.1319A>G	p.(Asp440Gly)														✓								✓							VUS
	RAD51D	5	c.412A>C	p.(Asn138His)														✓								✓							VUS
	RAD51D	**6**	**c.526T>C**	**p.(Phe176Leu)**							✓							✓								✓							VUS
	ATM	60	c.8734A>G	p.(Arg2912Gly)					✓	✓						✓	✓	✓								✓				✓			VUS
	ATM	22	c.3256C>T	p.(Arg1086Cys)													✓	✓								✓							VUS
	CHEK2	5	c.608A>G	p.(Asp203Gly)					✓		✓							✓								✓				✓			VUS
	TP53	5	c.461G>A	p.(Gly154Asp)						✓								✓															VUS
Other cancer susceptibility genes	*CDH1*	2	c.160A>G	p.(Arg54Gly)														✓								✓							VUS
	BRIP1	7	c.797C>T	p.(Thr266Met)														✓								✓							VUS
	MSH2	1	c.182A>C	p.(Gln61Pro)														✓								✓					✓		Likely Benign
	MSH6	4	c.1729C>T	p.(Arg577Cys)					✓									✓								✓				✓			Likely Benign
	NF1	**9**	**c.901G>A**	**p.(Asp301Asn)**							✓						✓	✓								✓							VUS
	NF1	**40**	**c.5953C>T**	**p.(Leu1985Leu)**							✓							✓															VUS
	PMS2	12	c.2068A>C	p.(Lys690Gln)														✓								✓							VUS
	PMS2	12	c.2012C>T	p.(Thr671Met)														✓								✓					✓		Likely Benign
	RAD51C	5	c.790G>A	p.(Gly264Ser)				✓								✓		✓				✓				✓				✓	✓		Likely Benign
	RAD51C	7	c.935G>A	p.(Arg312Gln)														✓								✓							VUS
	STK11	**8**	**c.1087A>C**	**p.(Thr363Pro)**							✓						✓	✓								✓							VUS
	FANCM	**21**	**c.5692G>A**	**p.(Val1898Met)**							✓							✓								✓							VUS

BC, Breast cancer; VUS, variants of uncertain clinical significance; novel variants in **bold**. Mutation nomenclature according to: *ATM* (LRG_135t1), *BARD1* (LRG_297t1), *BRIP1* (LRG_300t1), *CDH1* (LRG_301t1), *CHEK2* (LRG_302t1), *FANCM* (LRG_502t1), *MSH2* (LRG_218t1), *MSH6* (LRG_219t1), *NF1* (LRG_214), *PALB2* (LRG_308t1), *PMS2* (LRG_161t1), *RAD51C* (LRG_314t1), *RAD51D* (LRG_516t1), *STK11* (LRG_319t1), *TP53* (LRG_321t1).

Table S4. In silico assessment of the structural, functional, stability and flexibility impact of the predicted deleterious VUS in BC susceptibility genes.

Gene	Variant	PROTEIN STABILITY											PROTEIN BINDING	DNA BINDING	PROTEIN STRUCTURE & FUNCTION				PROTEIN FLEXIBILITY
		†FX	†I-Mutant3.0		†MU pro	†CUPSAT	†PoP MuSiC	†Dyna Mut	†ENCoM	†DUET	†SDM 2.0	†mCSM			SAAPdap/ SAAPpred	Missense 3D	SuSPect	SNAP2	**DynaMut
			PDB	Seq															
PALB2	c.3428T>A p.(Leu1143His	0.66	-1.44	*-1.75*	*-2.06*	*-3.54*	-0.13 61.07% RSA	1.36	3.16	-0.31	-0.12	-0.31	0.31	0.56	No structural damage	No structural damage	Score: 37<br>1. Low predicted RSA<br>2. His is less favourable than Leu in the PSSM	39	*-3.95*
BARD1	c.2189A>C p.(Gln730Pro)	*2.06*	-1.13	-0.37	-0.93	*-1.61*	1.45 3.14% RSA	-0.86	-0.37	-1.12	*-1.91*	-0.98	-0.96	0.18	*1. Disruption of a H-H bond.<br>2. Clash with surrounding AAs.*	*1. Buried Proline<br>2. Disruption of all SC:SC and/or SC:MC H-bonds formed by the WT aa*	Score: 48<br>1. Pro may destabilise secondary structure.<br>2. Pro is less favourable than Gln in the PSSM.	*73*	0.46
BARD1	c.1319A>G p.(Asp440Gly)	*4.17*	-1.12	-1.00	*-1.75*	-0.05	1.03 40.26% RSA	0.41	-0.05	-0.87	-0.06	-0.93	-0.38	1.40	No structural damage	No structural damage	*Score: 76<br>1. Position 38 of PF12796 (Ank2) domain<br>2. Low predicted RSA.<br>3. Gly is less favourable than Asp in the PSSM.*	*74*	0.06
RAD51D	c.412A>C p.(Asn138His)	0.02	N/A	-0.85	-1.27	*-2.43*	N/A	1.09	0.54	-0.48	0.42	-0.73	0.40	0.05	N/A	No structural damage	Score: 14<br>1. Position 81 of PF08423 (Rad51) domain.<br>2. High predicted RSA<br>3. His is more favourable than Asn in the PSSM.	-37	*-0.67*
RAD51D	**c.526T>C p.(Phe176Leu)**	0.21	N/A	-1.06	-0.63	-0.43	N/A	-0.59	-0.42	-0.45	0.57	-0.75	-1.42	0.19	N/A	No structural damage	Score: 10<br>1. Position 116 of PF08423 (Rad51) domain.<br>2. High predicted RSA.<br>3. Leu is less favourable than Phe in the PSSM.	-13	0.52
ATM	c.8734A>G p.(Arg2912Gly)	-6.52	*-1.96*	-1.42	*-1.83*	*-3.76*	*2.51 10.72% RSA*	-0.21	-0.92	0.00	-0.91	-1.08	-1.32	*-2.31*	No structural damage	*1. Buried charged Arg (RSA 2.4%) to uncharged Gly<br>2. Disruption of all SC:SC and/or SC:MC H-bonds formed by the WT aa*	*Score: 88<br>1. AA 181 of PF00454 (PI3_PI4_kinase) domain<br>2. Gly is less favourable than Arg in the PSSM.*	*81*	1.15
ATM	c.3256C>T p.(Arg1086Cys)	0.41	-0.45	-0.91	-0.33	0.88	*1.53 23.23% RSA*	1.30	2.72	0.00	-0.64	-0.26	0.18	-0.95	No structural damage	No structural damage	Score: 32<br>Low predicted RSA	15	*-4.65*
CHEK2	c.608A>G p.(Asp203Gly)	*2.38*	-1.24	*-1.62*	*-2.59*	*-4.23*	1.49 24.63% RSA	-0.06	0.12	-0.59	0.42	-1.00	-0.36	-0.05	*1. Disruption of a H-H bond.<br>2. Involved in binding<br>3. Interface aa*	No structural damage	Score: 40<br>1. Gly is less favourable than Asp in the PSSM.	*69*	*-0.15*
TP53	c.461G>A p.(Gly154Asp)	*3.76*	-0.71	-1.03	-0.18	*-2.77*	1.35 49.86% RSA	-1.13	0.02	*-1.53*	*-4.12*	-0.92	0.42	*-1.93*	N/A	*Disallowed phi/psi alert. The phi/psi angles are in favored region for WT aa but outlier region for MT aa.*	*Score: 98<br>1. Position 60 of PF00870 (P53 DNA-binding) domain.<br>2. Involved in DNA binding<br>3. Loss of Gly may affect secondary structure.*	*56*	*-0.03*

AA, amino acid; BC, Breast cancer; FX, FoldX; H-H, hudrogen bond; MT, mutant; PSSM, Position-Specific Scoring Matrix; RSA, relative solvent accessibility; Seq, sequence; WT, wild-type; novel variants in **bold**; predicted deleterious scores in *italics*. Mutation nomenclature according to: *ATM* (LRG_135t1), *BARD1* (LRG_297t1), *CHEK2* (LRG_302t1), *PALB2* (LRG_308t1), *RAD51D* (LRG_516t1), *TP53* (LRG_321t1). † ΔΔG (kcal/mol); * PDB ID or template-based predicted 3D structure; ** ΔΔSVib: kcal.mol-1.K-.

Table S5. In silico assessment of the structural, functional, stability and flexibility impact of the predicted deleterious VUS in other cancer susceptibility genes.

Gene	Variant	PROTEIN STABILITY											PROTEIN BINDING	DNA BINDING	PROTEIN STRUCTURE & FUNCTION				PROTEIN FLEXIBILITY
		†FX	†I-Mutant3.0		†MUpro	†CUP SAT	†PoP MuSiC	†Dyna Mut	†ENCoM	†DUET	†SDM2.0	†mCSM			SAAPdap/ SAAPpred	missense3D	SuSPect	SNAP2	**DynaMut
			PDB	Seq															
CDH1	c.160A>G<br>p.(Arg54Gly)	-0.33	N/A	*-1.65*	-1.33	0.33	N/A	N/A	N/A	0.40	1.65	-0.14	-0.36	-0.78	N/A	No structural damage	*Score: 93<br>1. Position 27 of PF08758 (Cadherin prodomain) domain<br>2. Gly is less favourable than Arg in the PSSM.*	*51*	N/A
BRIP1	c.797C>T<br>p.(Thr266Met)	-0.43	N/A	-0.18	-0.32	1.13	N/A	1.92	0.45	0.60	1.42	0.07	-0.48	*-2.18*	N/A	No structural damage	Score: 47<br>1. Position 19 of PF06733 (DEAD_2) domain<br>2. Residue predicted to be phosphorylated	8	*-0.57*
MSH2	c.182A>C<br>p.(Gln61Pro)	-0.56	-0.01	-0.56	-0.88	-0.17	-1.03<br>59.60%<br>RSA	0.27	0.29	0.20	-0.47	0.06	0.15	-0.34	*1. Involved in binding<br>2. Located in an interface*	No structural damage	*Score: 75<br>1. Position 45 of PF01624 (MutS_I) domain<br>2. Pro may destabilise secondary structure.*	-32	*-0.37*
MSH6	c.1729C>T<br>p.(Arg577Cys)	0.85	-0.81	-1.22	-1.33	*-1.68*	0.56<br>44.94%<br>RSA	1.60	2.16	0.15	0.05	0.14	-0.96	0.24	*Located in an interface*	No structural damage	Score: 38<br>1. High predicted RSA.	24	*-2.7*
NF1	**c.901G>A<br>p.(Asp301Asn)**	-0.29	N/A	-0.91	-1.11	-0.24	N/A	N/A	N/A	-0.50	0.49	-0.90	0.09	2.31	N/A	No structural damage	Score: 29<br>1. High predicted RSA.<br>2. Asn is less favourable than Asp in the PSSM.	23	N/A
NF1	**c.5953C>T<br>p.(Leu1985Leu)**	N/A	N/A	N/A	N/A	N/A	N/A	N/A	N/A	N/A	N/A	N/A	N/A	N/A	N/A	N/A	N/A	N/A	N/A
PMS2	c.2012C>T<br>p.(Thr671Met)	-0.65	N/A	-0.04	-0.53	-1.27	N/A	0.09	0.01	0.36	0.62	0.05	0.01	-0.84	N/A	No structural damage	Score: 14<br>Met is more favourable than Thr in the PSSM.	35	*-0.02*
PMS2	c.2068A>C<br>p.(Lys690Gln)	0.46	N/A	-0.73	-0.85	-1.48	N/A	-0.52	-0.79	-0.21	-0.69	-0.31	-0.15	-0.63	N/A	No structural damage	Score: 11<br>1. Position 17 of PF08676 (MutL_C terminal dimerisation) domain<br>2. The amino acid is at an interaction site.<br>3. Gln is more favourable than Lys in the PSSM.	7	0.99
RAD51C	c.790G>A<br>p.(Gly264Ser)	-0.58	N/A	-0.87	-0.78	1.97	N/A	0.04	-0.01	-0.78	-0.40	-1.09	-0.67	0.68	N/A	No structural damage	Score: 21<br>1. Position 167 of PF08423 (Rad51) domain.<br>2. Loss of Gly may affect secondary structure.<br>3. Ser is more favourable than Gly in the PSSM.	-3	0.02
RAD51C	c.935G>A<br>p.(Arg312Gln)	1.30	N/A	-0.75	-0.55	*-4.29*	N/A	-1.23	-0.27	*-1.58*	*-1.89*	-1.3	0.39	-0.77	N/A	*Buried charged aa (RSA 6.0%) to uncharged aa*	*Score: 81<br>1. Position 215 of PF08423 (Rad51) domain<br>2. Low predicted RSA.<br>3. Gln is less favourable than Arg in the PSSM.*	*90*	0.34
STK11	**c.1087A>C<br>p.(Thr363Pro)**	*7.04*	N/A	-0.55	-1.46	*-4.64*	N/A	-1.37	-0.19	*-1.81*	-1.01	*-1.56*	-0.50	-1.19	N/A	*1. Introduction of a buried Proline.<br>2. Disallowed phi/psi alert. The phi/psi angles are in favored region for WT residue but outlier region for MT aa.*	*Score: 71<br>1. This position is annotated as MOD_RES.<br>2. Residue predicted to be phosphorylated<br>3. Pro may destabilise secondary structure.<br>4. Low predicted RSA.<br>5. Pro is less favourable than Thr in the PSSM.*	-30	0.24
FANCM	**c.5692G>A<br>p.(Val1898Met)**	*2.16*	*-1.77*	-0.89	-0.91	-3.33	1.11<br>0.39%<br>RSA	-0.08	2	-1.46	*-2.07*	-1.11	-0.98	0.03	No structural damage	No structural damage	*Score: 57<br>1. Low predicted RSA.*	*69*	*-2.5*

																	2. Met is less favourable than Val in the PSSM.		

AA, amino acid; BC, Breast cancer; FX, FoldX; H-H, hudrogen bond; MT, mutant; PSSM, Position-Specific Scoring Matrix; RSA, relative solvent accessibility; Seq, sequence; WT, wild-type; novel variants in **bold;** predicted deleterious scores in *italics.* Mutation nomenclature according to: *BRIP1* (LRG_300t1), *CDH1* (LRG_301t1), *FANCM* (LRG_502t1), *MSH2* (LRG_218t1), *MSH6* (LRG_219t1), *NF1* (LRG_214), *PMS2* (LRG_161t1), *RAD51C* (LRG_314t1), *STK11* (LRG_319t1). † ΔΔG (kcal/mol); * PDB ID or template-based predicted 3D structure; ** ΔΔSVib: kcal.mol-1.K-

Table S6. TruSight Cancer (Illumina) target genes in alphabetical order.

AIP	*ALK*	*APC*	*ATM*	*BAP1*	*BLM*	*BMPR1A*	*BRCA1*	*BRCA2*	*BRIP1*
BUB1B	*CDC73*	*CDH1*	*CDK4*	*CDKN1C*	*CDKN2A*	*CEBPA*	*CEP57*	*CHEK2*	*CYLD*
DDB2	*DICER1*	*DIS3L2*	*EGFR*	*EPCAM*	*ERCC2*	*ERCC3*	*ERCC4*	*ERCC5*	*EXT1*
EXT2	*EZH2*	*FANCA*	*FANCB*	*FANCC*	*FANCD2*	*FANCE*	*FANCF*	*FANCG*	*FANCI*
FANCL	*FANCM*	*FH*	*FLCN*	*GATA2*	*GPC3*	*HNF1A*	*HRAS*	*KIT*	*MAX*
MEN1	*MET*	*MLH1*	*MSH2*	*MSH6*	*MUTYH*	*NBN*	*NF1*	*NF2*	*NSD1*
PALB2	*PHOX2B*	*PMS1*	*PMS2*	*PRF1*	*PRKAR1A*	*PTCH1*	*PTEN*	*RAD51C*	*RAD51D*
RB1	*RECQL4*	*RET*	*RHBDF2*	*RUNX1*	*SBDS*	*SDHAF2*	*SDHB*	*SDHC*	*SDHD*
SLX4	*SMAD4*	*SMARCB1*	*STK11*	*SUFU*	*TMEM127*	*TP53*	*TSC1*	*TSC2*	*VHL*
WRN	*WT1*	*XPA*	*XPC*						
